# Pulmonary Nontuberculous Mycobacteria, Ontario, Canada, 2020

**DOI:** 10.3201/eid2907.230216

**Published:** 2023-07

**Authors:** Theodore K. Marras, Paul Nelson, Adriana Peci, Melissa Richard-Greenblatt, Sarah Brode, Ashleigh Sullivan, Frances B. Jamieson, Julianne V. Kus

**Affiliations:** University of Toronto, Toronto, Ontario, Canada (T.K. Marras, M. Richard-Greenblatt, S. Brode, F.B. Jamieson, J.V. Kus);; University Health Network and Mount Sinai Hospital, Toronto (T.K. Marras, S. Brode);; Public Health Ontario, Toronto (P. Nelson, A. Peci, M. Richard-Greenblatt, A. Sullivan, F.B. Jamieson, J.V. Kus);; University of Oxford, Oxford, UK (M. Richard-Greenblatt);; West Park Healthcare Centre, Toronto (S. Brode)

**Keywords:** pulmonary nontuberculous mycobacteria, tuberculosis and other mycobacteria, mycobacteria, bacteria, atypical mycobacteria, epidemiology, Mycobacterium avium-intracellulare complex, Mycobacterium intracellulare, nontuberculous mycobacteria, respiratory infections, prevalence, Ontario, Canada

## Abstract

We measured annual prevalence of microbiologically defined nontuberculous mycobacterial lung disease in Ontario, Canada. *Mycobacterium avium* prevalence was 13 cases/100,000 persons in 2020, a 2.5-fold increase from 2010, indicating a large increase in true *M. avium* lung disease. During the same period, *M. xenopi* decreased nearly 50%, to 0.84 cases/100,000 persons.

Nontuberculous mycobacterial (NTM) pulmonary disease (NTM-PD) is increasingly common and difficult to manage (*1*). Thus, understanding its epidemiology is essential but challenging because the diagnosis requires microbiological, radiological, and clinical information (*1*). Because NTM-PD is not reportable in most jurisdictions, data required to study its epidemiology are generally unavailable. Many investigators have thus solely used microbiological criteria as a surrogate disease definition (*2*). We previously observed large increases in *Mycobacterium avium* complex (MAC) isolation in Ontario, Canada, during 1998–2010 (*3*). We report more recent prevalence of NTM-PD in Ontario.

## The Study

This retrospective cross-sectional study of persons in Ontario who had pulmonary NTM isolates during 2020 used Public Health Ontario’s laboratory records, which capture ≈95% of NTM in Ontario. We prepared cultures by using BACTEC MGIT 960 (Becton Dickinson, https://www.bd.com) and Lowenstein-Jensen slants. We used matrix-assisted laser desorption/ionization time-of-flight mass spectrometry, line-probe assays (GenoType; Hain Lifescience, https://www.hain-lifescience.de), a laboratory-developed MAC real-time PCR, or 16S rDNA sequencing to identify NTM to species/subspecies levels.

**Figure 1:**
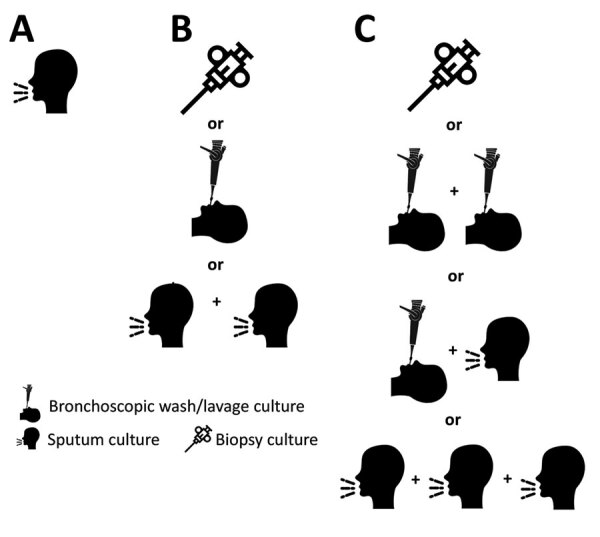
Categories of nontuberculous mycobacteria pulmonary disease, Ontario, Canada, 2020. A) Uncertain state: 1 sputum culture. B) Standard microbiological criteria: >2 sputum cultures with the same species, or 1 bronchoscopic or lung biopsy culture. C) Strict microbiological criteria (subset of standard): >3 sputum cultures with the same species, or >2 bronchoscopic cultures with the same species, or 1 bronchoscopic plus 1 sputum culture with the same species, or 1 lung biopsy culture. Some images were obtained from Flaticon (https://www.flaticon.com).

Lacking clinical information, we defined surrogate NTM-PD criteria microbiologically, in 3 categories: uncertain (single sputum), standard (guidelines’ microbiological definition [*1*]), and strict (additional culture required) (Figure 1). Although standard microbiological criteria demonstrated a 70%–100% positive predictive value (*3*), we created the strict category (subset of standard) because it is unknown whether the diagnostic test characteristics might vary by potentially changing levels of environmental NTM exposure, possibly affecting frequencies of specimen contamination or colonization. Patients who had NTM isolated in 2020 had previous 24-month sample histories reviewed to determine if they reached disease thresholds. W used Statistics Canada population data for calculating prevalence and age- and sex-standardization (https://www150.statcan.gc.ca/t1/tbl1/en/tv.action?pid = 1710000501). We performed analysis by using SAS Enterprise Guide 9.4 (SAS Institute, https://www.sas.com). The Public Health Ontario Ethics Review Board approved this study.

During 2020, the population of Ontario was 14,726,022, and of 41,471 pulmonary samples tested for mycobacteriology, 8,412 (20.3%) grew NTM. All-species prevalence (cases/100,000 population) by NTM-PD definition was as follows: uncertain, 12.9 (n = 1,899); standard, 19.2 (n = 2,824); and strict, 10.9 (n = 1,602), (Table 1, by species). Overall cases of standard (69.2%) and strict (73.1%) microbiological disease were caused mostly by *M. avium* (69.2% and 73.1%), followed by *M. intracellulare* (6.4% and 6.3%), *M .abscessus* (5.8% and 7.1%), and *M. xenopi* (4.4% and 3.9%).

**Table 1:** Characteristics of pulmonary nontuberculous mycobacterial disease, by microbiological definition, among patients in Ontario, Canada, 2020*

NTM species/subspecies	Standard definition†			Strict definition‡	
	No. (%) patients	Prevalence§		No. (%) patients	Prevalence§
Common MAC species					
* M. avium*	1,954 (69.2)	13.27		1,171 (73.1)	7.95
* M. intracellulare*	182 (6.4)	1.24		101 (6.3)	0.69
* M. chimaera*	39 (1.4)	0.26		18 (1.1)	0.12
*M.* *abscessus* and its subspecies					
* M. abscessus* (total)	164 (5.8)	1.11		114 (7.1)	0.77
subspecies *abscessus*	95 (3.4)	0.65		66 (4.1)	0.45
subspecies *bolletii*	1 (0.04)	0.01		1 (0.06)	0.01
subspecies *massiliense*	52 (1.8)	0.35		38 (2.4)	0.26
subspecies unassigned	16 (0.6)	0.11		9 (0.6)	0.06
Other species					
* M. xenopi*	123 (4.4)	0.84		63 (3.9)	0.43
* M. fortuitum*	98 (3.5)	0.67		53 (3.3)	0.36
* M. gordonae*	117 (4.1)	0.79		26 (1.6)	0.18
Other NTM species	147 (5.2)	1.00		56 (3.5)	0.38
Total	2,824 (100)	19.0		1,602 (100)	10.9

*Counts within each disease definition represent unique patients; some patients fulfilled criteria for >1 species. MAC, *Mycobacterium avium* complex; NTM, nontuberculous myobacteria. †Standard microbiological criteria: >2 sputum cultures with the same species, or 1 bronchoscopic or lung biopsy culture. ‡Strict microbiological criteria (subset of standard): >3 sputum cultures with the same species, or >2 bronchoscopic cultures with the same species, or 1 bronchoscopic plus 1 sputum culture with the same species, or 1 lung biopsy culture. §Cases per 100,000 population.

More female than male patients were classified with standard disease (1,507 [53.4%] vs. 1,285 [45.5%]) and strict disease (892 [55.7%] vs. 696 [43.4%]) (Table 2). Substantial discrepancies in sex ratio from parity were seen for *M. avium*, *M. intracellulare*, and *M. abscessus*, favoring female patients, and *M. gordonae*, favoring male patients. Adjusting for population sex distribution did not greatly alter those observations.

**Table 2:** Patients with pulmonary NTM, by microbiological definition, *Mycobacterium* species, and patient sex, Ontario, Canada, 2020*

NTM species	Standard definition, no. (%) patients†				Strict definition, no. (%) patients‡		
	Female	Male	Unknown		Female	Male	Unknown
*M. avium*	1,051 (53.8)	881 (45.1)	22 (1.1)		653 (55.8)	506 (43.2)	12 (1.0)
*M. intracellulare*	114 (62.6)	65 (35.7)	3 (1.6)		71 (70.3)	28 (27.7)	2 (2.0)
*M. chimaera*	17 (43.6)	22 (56.4)	0		10 (55.6)	8 (44.4)	0
*M. abscessus*	102 (62.1)	62 (37.8)	0		68 (59.6)	46 (40.4)	0
*M. xenopi*	63 (51.2)	57 (46.3)	3 (2.4)		29 (46.0)	34 (54.0)	0
*M. fortuitum*	47 (48.0)	50 (51.0)	1 (1.0)		26 (49.1)	27 (50.9)	0
*M. gordonae*	46 (39.3)	70 (59.8)	1 (0.9)		10 (38.5)	16 (61.5)	0
Other NTM	67 (45.6)	78 (53.1)	2 (1.4)		25 (44.6)	31 (55.4)	0
Total	1,507 (53.4)	1,285 (45.5)	32 (1.1)		892 (55.7)	696 (43.4)	14 (0.9)

*Counts within each disease definition represent unique patients; some patients fulfilled criteria for >1 species. NTM, nontuberculous mycobacteria. †Standard microbiological criteria: >2 sputum cultures with the same species, or 1 bronchoscopic or lung biopsy culture ‡Strict microbiological criteria (subset of standard): >3 sputum cultures with the same species, or >2 bronchoscopic cultures with the same species, or 1 bronchoscopic plus 1 sputum culture with the same species, or 1 lung biopsy culture.

Depending on species, patients who were ≥60 years of age comprised 63%–85% (74.2% overall) of those with standard disease and 61%–85% (76.9% overall) of those with strict disease (Appendix Table 1). Younger patients generally represented small minorities. Age-standardized prevalence ratio for all species combined increased from 0.05 in the youngest age group to 4.46 in the oldest age group among patients meeting standard criteria and from 0.07 to 4.56 among patients meeting strict criteria.

**Figure 2:**
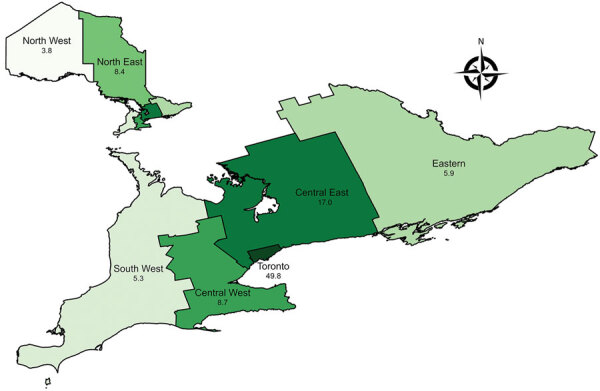
Prevalence of nontuberculous mycobacterial pulmonary disease (standard definition) by Ontario health region, Ontario, Canada, 2020. Numbers below region names indicate number of cases per 100,000 population.

We found striking regional heterogeneity in the frequency of NTM-PD (Figure 2; Appendix Table 2). Prevalence of all-species NTM-PD, by standard and strict definitions was lowest in the North West region (3.8 vs. 2.9 cases/100,000 persons) and highest in Toronto (49.8 vs. 28.8 cases/100,000 persons). We compiled selected comparisons between nonoverlapping groups (exclusively standard versus strict definition patients) (Appendix Tables 3, 4).

## Conclusions

In this population-based study of NTM-PD in Ontario, we observed high levels by using standard (19.0 cases/100,000 persons) and strict (10.9 cases/100,000 persons) microbiological criteria. *M. avium* comprised most of cases, followed distantly by *M. intracellulare* and *M. abscessus* and less commonly *M. xenopi*. The total NTM-PD prevalence (standard definition) of 19.0 cases/100,000 persons was nearly double the previously reported 9.8 cases/100,000 persons in 2010 (*3*). During the same period, *M. xenopi* prevalence decreased by nearly half, from 1.54 to 0.84 cases/100,000 persons, and *M. abscessus* increased 3.7-fold, from 0.30 to 1.11 cases/100,000 persons (*3*). In our previous study, *M. avium* was not separated from other MAC species, but ≈85% of contemporary Ontario MAC isolates were *M. avium* (*4*). The prevalence of *M. avium* pulmonary disease in Ontario, by standard microbiological definition, increased 2.5-fold, from 5.34 cases/100,000 persons in 2010 (*3*) to 13.27 cases/100,000 persons in 2020, explaining virtually all of the NTM-PD increase.

Historically, most population-based studies (*2*) and a recent large managed care study in the United States (*5*) have shown increases in NTM-PD. Trends in NTM species frequencies were reviewed in 8 recent population-based studies that had species-level data (*6**‒**13*). In Denmark (1997–2008) (*6*) and Madrid, Spain (2013–2017) (*7*), there were no convincing trends. In Croatia (2006–2010), *M. fortuitum* increased (*8*). The remaining 5 studies demonstrated large increases in MAC (Barcelona/Catalonia in 1994–2014 [*9*]; Queensland, Australia, in 1999–2005 [*10*]; and the United Kingdom in 2007–2012 [*11*]) or specifically *M. avium* (the Netherlands in 2000–2006 [*12*] and Belgium in 2007–2016 [*13*]), without major changes in other species. The Ontario experience is similar, with the exception of the reduction in *M. xenopi*. Most other regions had increases in other species in addition to *M. avium* or MAC. The marked reduction in *M. xenopi* in this study is unexplained but might be related to the increase in *M. avium*. *M. avium* might be outcompeting *M. xenopi* in a common environmental niche or overgrowing the slow-growing *M. xenopi* in culture. 

The main limitation of our study is the lack of clinical and radiological information. Defining NTM-PD solely on microbiological criteria over-estimates prevalence by misclassifying persons who fulfil microbiological criteria but not clinical/radiological criteria. Although microbiological criteria have good positive predictive values, the diagnostic test characteristics might be unstable (e.g., if there is a major change in NTM exposure). As suggested by hospital NTM pseudo-outbreaks (*14*), increased environmental NTM exposure might increase colonization or contamination of oropharyngeal and airway mucosal secretions and thereby specimens submitted for mycobacterial testing, thus reducing the positive predictive value of microbiological criteria for NTM-PD.

Given our uncertainties, we chose standard and strict for disease definitions, rather than previously used terms such as definite. Regardless, we believe that an increase in environmental NTM exposure, probably contributing to our large increase in *M. avium* isolation, would eventually cause increased disease. There were no changes in laboratory methods explaining the observed *M. avium* increase, and there was no consistent increase in contemporary specimen submission (increased testing bias). Given the absence of laboratory changes, we believe that the increase is related to increased environmental exposures, resulting in increased colonization and disease, or better patient identification for testing by clinicians. Concerning better patient identification for testing, the proportion of culture-positive pulmonary specimens increased from ≈10% in 2010 to 20.3% in 2020 (18.0% in 2018 and 19.3% in 2019). There was a near-linear increase in chest computed tomography (CT) scanning in Ontario during 2007–2016 (*15*), possibly increasing detection because CT scans identify characteristic findings of NTM, accurately driving clinical suspicion. Increased CT use probably contributes to greater detection of true disease, although it could not explain the reduction in *M. xenopi*.

In summary, this study identified a large increase in the prevalence of microbiologically defined *M. avium* lung disease in this region, undoubtedly indicating a large increase in the prevalence of true lung disease caused by *M. avium*. Clinicians should be aware of the causes of this increase and investigators should determine to what extent the increase in microbiologically defined disease reflects true disease.

Appendix Additional information on pulmonary nontuberculous mycobacteria, Ontario, Canada, 2020.
