# Supplementary material for: Pulmonary Nontuberculous Mycobacteria, Ontario, Canada, 2020
Source: Emerg Infect Dis. 2023 Jul;29(7):1415–9. doi: 10.3201/eid2907.230216 (PMC10310396; doi:10.3201/eid2907.230216)
Supplement: Appendix — Additional information on pulmonary nontuberculous mycobacteria, Ontario, Canada, 2020. [file 23-0216-Techapp-s1.pdf]

Article DOI: https://doi.org/10.3201/eid2907.230216

EID cannot ensure accessibility for supplementary materials supplied by authors. Readers who have difficulty accessing supplementary content should contact the authors for assistance.

Pulmonary Nontuberculous Mycobacteria, Ontario, Canada, 2020

Appendix

Appendix Table 1. Number and percent of nontuberculous mycobacterial species by age group of patients in the microbiological standard and strict disease state, Ontario, 2020*

	Standard definition age groups (years) †‡					
NTM Species	0-19	20-39	40-59	60-69	70-79	80+
M. avium (n=1,954)	15 (0.8%)	153 (7.8%)	321 (16.4%)	508 (26.0%)	555 (28.4%)	401 (20.5%)
M. intracellulare (n=182)	1 (0.5%)	7 (3.8%)	20 (11.0%)	37 (20.3%)	66 (36.3%)	51 (28.0%)
M. chimaera (n=39)	1 (2.6%)	2 (5.1%)	10 (25.6%)	11 (28.2%)	8 (20.5%)	7 (17.9%)
M. abscessus (n=164)	12 (7.3%)	31 (18.9%)	18 (11.0%)	32 (19.5%)	51 (31.1%)	20 (12.2%)
M. xenopi (n=123)	0 (0.0%)	6 (4.9%)	18 (14.6%)	42 (34.1%)	39 (31.7%)	18 (14.6%)
M. fortuitum (n=98)	0 (0.0%)	3 (3.1%)	23 (23.5%)	26 (26.5%)	27 (27.6%)	19 (19.4%)
M. gordonae (n=117)	1 (0.9%)	12 (10.3%)	24 (20.5%)	26 (22.2%)	28 (23.9%)	26 (22.2%)
Other NTM (n=147)	1 (0.7)	24 (16.3%)	25 (17.0%)	43 (29.3%)	31 (21.1%)	23 (15.6%)
Total (n=2,824)	31 (1.1%)	238 (8.4%)	459 (16.3%)	725 (25.7%)	805 (28.5%)	565 (20.0%)
Total age standardized prevalence ratio	0.05	0.30	0.61	2.14	3.69	4.46
	Strict definition age groups (years) §‡					
	0-19	20-39	40-59	60-69	70-79	80+
M. avium (n=1,171)	13 (1.1%)	74 (6.3%)	172 (14.7%)	303 (25.9%)	351 (30.0%)	257 (21.9%)
M. intracellulare (n=101)	1 (1.0%)	3 (3.0%)	11 (10.9%)	23 (22.8%)	40 (39.6%)	23 (22.2%)
M. chimaera (n=18)	0 (0%)	1 (5.6%)	5 (27.8%)	3 (16.7%)	6 (33.3%)	3 (16.7%)
M. abscessus (n=114)	8 (7.0%)	24 (21.1%)	13 (11.4%)	23 (20.2%)	35 (30.7%)	11 (9.6%)
M. xenopi (n=63)	0 (0.0%)	2 (3.2%)	9 (14.3%)	23 (36.5%)	18 (28.6%)	11 (17.5%)
M. fortuitum (n=53)	0 (0.0%)	1 (1.9%)	10 (18.9%)	16 (30.2%)	18 (34.0%)	8 (15.1%)
M. gordonae (n=26)	0 (0.0%)	3 (11.5%)	1 (3.8%)	7 (26.9%)	6 (23.1%)	9 (34.6%)
Other NTM (n=56)	1 (1.8%)	6 (10.7%)	11 (19.6%)	22 (39.3%)	11 (19.6%)	5 (8.9%)
Total	23 (1.4%)	114 (7.1%)	232 (14.5%)	420 (26.2%)	485 (30.3%)	327 (20.4%)
Total age standardized prevalence ratio	0.07	0.25	0.55	2.19	3.93	4.56

*Counts within each disease definition represent unique patients; some patients fulfilled criteria for >1 species.
†Standard microbiological criteria: ≥2 sputum cultures with the same species, or 1 bronchoscopic or lung biopsy culture
‡Age unknown for 1 (<0.1%) person each with standard and strict microbiological disease respectively
§Strict microbiological criteria (subset of standard): ≥3 sputum cultures with the same species, or ≥2 bronchoscopic cultures with the same species, or 1 bronchoscopic plus 1 sputum culture with the same species, or 1 lung biopsy culture.

Appendix Table 2. Prevalence per 100,000 population and number of cases of nontuberculous mycobacterial pulmonary disease per standard definition* by species and health region in Ontario, 2020

Health Region	*M. avium*	*M. intracellulare*	*M. abscessus*	Other Species	All Species
North West	2.5 (6)	0.4 (1)	0.4 (1)	0.4 (1)	3.8 (9)
North East	5.0 (28)	1.6 (9)	0.5 (3)	1.3 (7)	8.4 (47)
Eastern	2.6 (51)	0.6 (12)	0.5 (10)	2.1 (41)	5.9 (114)
Central East	10.8 (486)	1.0 (47)	1.5 (66)	3.6 (163)	17.0 (762)
Toronto	38.3 (1,195)	2.0 (62)	1.9 (60)	7.6 (237)	49.8 (1,554)
Central West	5.3 (151)	1.1 (32)	0.7 (20)	1.6 (45)	8.7 (248)
South West	2.2 (37)	1.1 (19)	0.2 (4)	1.8 (30)	5.3 (90)
Total Ontario	13.1 (1,954)	1.2 (182)	1.1 (164)	3.5 (524)	19.0 (2,824)

*Standard microbiological criteria: ≥2 sputum cultures with the same species, or 1 bronchoscopic or lung biopsy culture.

Appendix Table 3. Number, percent and prevalence per 100,000 of pulmonary nontuberculous mycobacterial disease, by non-overlapping microbiological definitions, Ontario, 2020*

NTM species/subspecies	Exclusively standard definition†	Strict definition‡
Common MAC species		
M. avium	783 (58.6%) / 5.32	1,171 (73.1%) / 7.95
M. intracellulare	81 (6.1%) / 0.55	101 (6.3%) / 0.69
M. chimaera	21 (1.6%) / 0.14	18 (1.1%) / 0.12
M. *abscessus* and its subspecies		
M. abscessus (total)	50 (8.5%) / 0.77	114 (7.1%) / 0.77
subspecies *abscessus*	29 (2.2%) / 0.20	66 (4.1%) / 0.45
subspecies *bolletii*	0 (0%) / 0	1 (0.06%) / 0.01
subspecies *massiliense*	14 (1.1%) / 0.10	38 (2.4%) / 0.26
subspecies unassigned	7 (0.5%) / 0.05	9 (0.6%) / 0.06
Other species		
M. xenopi	60 (4.5%) / 0.41	63 (3.9%) / 0.43
M. fortuitum	45 (3.4%) / 0.31	53 (3.3%) / 0.36
M. gordonae	91 (6.8%) / 0.62	26 (1.6%) / 0.18
Other NTM species	91 (6.8%) / 0.62	56 (3.5%) / 0.38
Total	1,336 (100%) / 9.07	1,602 (100%) / 10.9

*Counts within each disease definition represent unique patients; some patients fulfilled criteria for >1 species.
†Exclusively standard microbiological criteria: 2 sputum cultures with the same species, or 1 bronchoscopic or lung biopsy culture
‡Strict microbiological criteria: ≥3 sputum cultures with the same species, or ≥2 bronchoscopic cultures with the same species, or 1 bronchoscopic plus 1 sputum culture with the same species, or 1 lung biopsy culture.

Appendix Table 4. Number of patients with pulmonary nontuberculous mycobacteria by non-overlapping microbiological definitions, *Mycobacterium* species and sex, Ontario, 2020*

	Exclusively standard definition†			Strict definition‡		
NTM Species	Female	Male	Unknown	Female	Male	Unknown
M. avium	398 (50.8%)	375 (47.9%)	10 (1.3%)	653 (55.8%)	506 (43.2%)	12 (1.0%)
M. intracellulare	43 (53.1%)	37 (45.7%)	1 (1.2%)	71 (70.3%)	28 (27.7%)	2 (2.0%)
M. chimaera	7 (33.3%)	14 (66.7%)	0	10 (55.6)	8 (44.4%)	0
M. abscessus	34 (68.0%)	16 (32.0%)	0	68 (59.6%)	46 (40.4%)	0
M. xenopi	34 (56.7%)	23 (38.3%)	3 (5.0%)	29 (46.0%)	34 (54.0)	0
M. fortuitum	21 (46.7%)	23 (51.1%)	1 (2.2%)	26 (49.1%)	27 (50.9%)	0
M. gordonae	36 (39.6%)	54 (59.3%)	1 (1.1%)	10 (38.5%)	16 (61.5%)	0
Other NTM	42 (46.2%)	47 (51.6%)	2 (2.2%)	25 (44.6%)	31 (55.4%)	0
Total	615 (50.3%)	589 (48.2%)	18 (1.5%)	892 (55.7%)	696 (43.4%)	14 (0.9%)

*Counts within each disease definition represent unique patients; some patients fulfilled criteria for >1 species.
†Exclusively standard microbiological criteria: 2 sputum cultures with the same species, or 1 bronchoscopic or lung biopsy culture
‡Strict microbiological criteria: ≥3 sputum cultures with the same species, or ≥2 bronchoscopic cultures with the same species, or 1 bronchoscopic plus 1 sputum culture with the same species, or 1 lung biopsy culture.
